# From Micro to Macro: Exploring Preference-Based Person Centered Care from Multiple Perspectives

**DOI:** 10.1093/geroni/igaa057.3062

**Published:** 2020-12-16

**Authors:** Katherine Abbott, Kristine Williams

## Abstract

Advancing our knowledge related to honoring nursing home resident preferences is a cornerstone of person-centered care (PCC). While there are multiple approaches to providing PCC, we focus on resident preferences as assessed via the Preferences for Everyday Living Inventory (PELI). The PELI is an evidenced-based, validated instrument that can be used to enhance the delivery of PCC. In this symposium, we explore the perspectives of a variety of stakeholders including nursing home residents, staff, and the impact of preference-based care on provider level regulatory outcomes. First, we present a comparative study of preference importance among n=317 African America and White nursing home residents that found more similarities than differences between the two groups. Second, a content analysis of the responses from n=196 interviews with nursing home residents details the barriers and facilitators connected to their levels of satisfaction with their preferences being fulfilled. Third, perspectives from n=27 direct care workers explore the concept of pervasive risk avoidance to the delivery of PCC. Fourth, systems-level practices, such as shift assignments and provider schedules are identified as barriers to successfully fulfilling resident preferences from the perspectives of n=19 staff within assisted living. Our final presentation utilizes a fixed-effects panel regression analysis with n=551 Ohio nursing home providers to explore the impact of PELI use on regulatory outcomes such as substantiated complaints and deficiency scores reported in the CMS Nursing Home Compare data. Discussant Dr. Kristi Williams will integrate findings, highlighting implications for policy, practice, and future directions. Research in Quality of Care Interest Group Sponsored Symposium.

